# Serum soluble fibrinogen‐like protein 2 concentration predicts delirium after acute pancreatitis

**DOI:** 10.1002/brb3.1261

**Published:** 2019-03-18

**Authors:** Wen‐Bin Xu, Qian‐Hua Hu, Chan‐Ni Wu, Zhi‐Kun Fan, Zhang‐Fa Song

**Affiliations:** ^1^ Department of General Surgery The Zhejiang Xiaoshan Hospital Hangzhou China; ^2^ Department of Gastroenterology The Zhejiang Xiaoshan Hospital Hangzhou China; ^3^ Department of Anorectal Surgery Sir Run Run Shaw Hospital, School of Medicine, Zhejiang University Hangzhou China

**Keywords:** acute pancreatitis, delirium, fibrinogen‐like protein 2, severity

## Abstract

**Objective:**

Inflammation can cause delirium. Soluble fibrinogen‐like protein 2 (sFGL2) is a modulator of the immune response and more recently found to be a biomarker for brain injury. This study was designed to discover the predictive capability of serum sFGL2 concentrations for delirium after acute pancreatitis (AP).

**Materials and Methods:**

In this prospective, observational study, serum sFGL2 concentrations were quantified in 184 healthy controls and in 184 AP patients. Disease severity was assessed by Acute Physiology and Chronic Health Care Evaluation II score, Ranson score, multiple organ dysfunction score, and sequential organ failure assessment score. Delirium was recorded during hospital stay. Predictors of delirium were identified using multivariate analysis.

**Results:**

Serum sFGL2 concentrations were substantially higher in AP patients than in controls. Serum sFGL2 concentrations were intimately correlated with the preceding severity parameters. Serum sFGL2 and the aforementioned severity parameters were independent predictors for delirium. Under receiver operating characteristic curve, the discriminatory ability of serum sFGL2 was equivalent to those of the above‐mentioned severity parameters. Moreover, serum sFGL2 dramatically improved the predictive value of the aforementioned severity parameters.

**Conclusions:**

Elevation of serum sFGL2 concentrations is strongly associated with the AP severity and has the potential to distinguish delirium after AP.

## 1 INTRODUCTION

Acute pancreatitis (AP) is a common type of inflammatory disease with deleterious local and systemic reaction, which is caused by the release of digestive enzymes to the pancreatic interstitium and to the systemic circulation (Frossard, Steer, & Pastor, 2008; Imrie, 1997; Soran, Chelluri, Lee, & Tisherman, 2000). The AP etiology is heterogeneous as well as its underlying mechanisms are rather complicated and involve a complex cascade of events, including inflammation, microcirculatory disturbances, and coagulation abnormalities (Gukovskaya, Gukovsky, Algül, & Habtezion, 2017; Habtezion, 2015; Hagiwara et al., 2009; Ranson, Lackner, Berman, & Schinella, 1977; Rawla, Bandaru, & Vellipuram, 2017; Singh & Garg, 2016; Zhou & Chen, 2002). In general, the Acute Physiology and Chronic Health Care Evaluation II (APCHCE II) score, multiple organ dysfunction score (MODS), Ranson score, and sequential organ failure assessment (SOFA) score have been broadly used for assessment of AP severity and prognosis (Marshall et al., 1995; Ranson & Pasternack, 1977; Ranson et al., 1974; Vincent et al., 1996). Delirium is a common complication in patients with severe diseases, which is associated with increasing mortality and morbidity. Its occurrence might be related to some pathophysiological processes, such as inflammation, microcirculatory disturbances, and oxidative reaction, therefore leading to brain injury (Cerejeira, Nogueira, Luís, Vaz‐Serra, & Mukaetova‐Ladinska, 2012; Neerland et al., 2016; Vasunilashorn et al., 2017). Such risk factors are apt to appear in AP (Gukovskaya et al., 2017; Habtezion, 2015; Hagiwara et al., 2009; Ranson et al., 1977; Rawla et al., 2017; Singh & Garg, 2016; Zhou & Chen, 2002). A great number of studies have assessed delirium in severe illnesses (Barbateskovic et al., 2016; Bhattacharya et al., 2017; Desforges & Lipowski, 1989; Desforges & Lipowski, 1989; Kratz, Heinrich, Schlauß, & Diefenbacher, 2015; Kumar, Jayant, Arya, Magoon, & Sharma, 2017; Li et al., 2018; Lundstrom, Edlund, Bucht, Karlsson, & Gustafson, 2003; Nie, Zhao, Zhang, Jiang, & Yang, 2012; Sanguineti, Wild, & Fain, 2014; Zhu et al., 2017). However, to the best of my knowledge, there are data unavailable on delirium solely in AP.

Fibrinogen‐like protein 2 (FGL2) belongs to the fibrinogen‐associated protein superfamily (Chan et al., 2003; Hancock et al., 2004; Su et al., 2008). FGL2 has two different forms, including FGL2 prothrombinase and soluble FGL2 (sFGL2), which have distinct properties. The former is the membrane‐protein form, which is mainly expressed on the reticuloendothelial cells and exerts procoagulative activity; the latter is the soluble form, which is constitutively expressed by CD4+ and CD8+ T cells and presents contradictory properties in tissue injuries (Chan et al., 2003; Marazzi et al., 1998; Shevach, 2009; Yuwaraj, Ding, Liu, Marsden, & Levy, 2001). Some studies have shown that sFGL2 levels in the peripheral blood were significantly elevated in some inflammatory or procoagulative diseases, such as acute cerebral ischemic‐reperfusion injury, renal ischemia reperfusion injury during kidney auto‐transplantation, hepatic cirrhosis with hepatocellular carcinoma, and inflammatory bowel disease (Melnyk et al., 2011; Shalev et al., 2009; Tang et al., 2017; Zhu et al., 2005). In AP rats or patients, FGL2 expressions of pancreatic tissues were significantly increased and its elevation was highly associated with progression of disease (Ye et al., 2013, 2014). Intriguingly, FGL2 can be produced from animal brain tissues after ischemic or hemorrhagic injury (Yuan et al., 2017, 2016; Zhang et al., 2015). Of note, in a recent study, serum FGL2 levels tended to strongly correlate with cerebral infarct size in rats with acute cerebral ischemic‐reperfusion injury (Zhang et al., 2015); more recently, serum FGL2 levels were prone to increase in severe traumatic brain injury and its elevation was highly relevant to clinical severity and mortality (Chen et al., 2018), indicating that FGL2 should be a promising biomarker for brain injury. To the best of my knowledge, it is unclear whether circulating sFGL2 is related to delirium after AP. The purpose of the current study was to determine serum sFGL2 levels and to further examine its association with the severity and delirium following AP.

## 2 MATERIALS AND METHODS

### 2.1 Study population

This is a prospective and observational study conducted on the Zhejiang Xiaoshan hospital, China. A group of first‐episode AP patients were consecutively recruited during the period of between February 2013 and February 2017. AP was diagnosed in accordance with the criteria proposed by the International Atlanta Symposium on Acute Pancreatitis in 2012 (Banks et al., 2013). We excluded pregnant AP women and those patients with less than 18 years of age, malignant diseases, previous systemic diseases, immunosuppressive disorders, inflammatory diseases within recent 1 month, a documented history of dementia, delirium or depressive illness, inability to speak or understand Chinese, any severe visual or auditory disorders, or previous neurological diseases like ischemic or hemorrhagic stroke, and severe head trauma. Also, we enrolled a group of controls free of systemic diseases, cancer, infection, and medications at study entrance. The study protocol was established according to World Medical Association Declaration of Helsinki and also approved by the ethics committee at our hospital. Written informed consent was required for participation.

### 2.2 Assessment

We recorded information as follows: demographic data (such as age and sex), body mass index (BMI), time period from onset of pain to hospital admission, time period from onset of pain to sample collection, etiologies, and treatment methods (conservative or operative treatment). The etiologies of AP comprised biliary, alcoholic, hypertriglyceremic, or others, which were identified according to the previous report (Khan, Nordback, & Sand, 2013). Forty‐eight–hour Ranson score, APACHE II score, MODS, and SOFA score were utilized to evaluate AP severity (Marshall et al., 1995; Ranson & Pasternack, 1977; Ranson et al., 1974; Vincent et al., 1996). Based on the Confusion Assessment Method (Ely, Inouye et al., 2001; Ely, Margolin et al., 2001), patients were evaluated for delirium twice daily until they were discharged from hospital.

### 2.3 Determination

We drew peripheral blood via the antecubital vein from the patients on admission and from the controls at study entrance. Some routine laboratory tests were carried out to gauge white blood cell (WBC) count and C‐reactive protein (CRP) level. We consecutively collected blood samples and the separated serum was preserved −80°C for further assay. In order to detect serum sFGL2 concentrations, we utilized enzyme‐linked immunosorbent assay (BioLegend Company, USA). The quantification was in batches done every 3 months according to the protocol of the manufacturer. Using the same equipment, all samples were in duplicates measured by the same laboratory technician without access to clinical information.

### 2.4 Statistical analysis

Statistical softwares used in the current study included SPSS 19.0 statistical software (SPSS Inc., Chicago, IL) and MedCalc 9.6.4.0 (MedCalc Software, Mariakerke, Belgium). The Kolmogorovor–Smirnov test or Shapiro–Wilk test was done to assess normality of continuous variables. Because all continuous variables showed nonnormal distribution, they were presented in the form of median (interquartile range [IQR]). Subsequently, the Mann–Whitney *U* test was carried out for two‐group comparison of continuous variables. Categorical data were reported as count (percentage) and their intergroup comparison was performed using the Chi‐square test or Fisher exact test. Bivariate correlation was analyzed using the Spearman's correlation test. The logistic regression model was configured to reveal independent predictors for delirium. Independent association was indicated by odds ratio (OR) and 95% confidence interval (CI) values. The receiver operating characteristic (ROC) curve was constructed. For predicting delirium, area under curve (AUC) and 95% CI values were calculated. Optimal cutoff values of serum sFGL2 levels were selected, as well as the corresponding sensitivity and specificity values were yielded. Using *Z* test, intergroup comparison was done for AUC. Also, the addictive effect of serum sFGL2 levels to other variables were assessed under combined Logistic regression model. *p* values <0.05 were considered statistically significant.

## 3 RESULTS

### 3.1 Study population characteristics

At first, a total of 264 AP patients were evaluated. For eligibility, 12 pregnant AP women were excluded and other 68 patients were also excluded because of the following reasons including less than 18 years of age (four cases), malignant diseases (eight cases), previous systemic diseases (12 cases), immunosuppressive disorders (seven cases), inflammatory diseases within recent 1 month (six cases), a documented history of dementia, delirium, or depressive illness (eight cases), inability to speak or understand Chinese (two cases), any severe visual or auditory disorders (four cases), previous neurological diseases like ischemic or hemorrhagic stroke and severe head trauma (12 cases), refusal to participation (two cases), and unavailable samples (three cases). Eventually, a total of 184 AP patients were assessed in the current study. Alternatively, a total of 184 healthy individuals constituted a control group. There were not statistically significant differences between the patients and the controls with respect to age and gender proportion.

This group of AP patients with 127 males and 57 females, ranged from 29 to 77 years at age (median, 53 years; IQR, 41–62 years). There was 26.2 kg/m^2^at the median BMI (range, 20.4–30.0 kg/m^2^; IQR, 23.9–27.8 kg/m^2^). Patients were hospitalized from 1.0 to 34.5 hr after the onset of pain (median, 18.0 hr; IQR, 13.1–23.8 hr). The median time for collection of peripheral blood was 19.4 hr (range, 1.2–37.2 hr; IQR, 13.8–26.4 hr). Analysis on AP etiology showed that biliary AP occurred in 60 patients; alcoholic, in 71 patients; hypertriglyceremic, in 37 patients; others, in 16 patients. For the AP therapy, 139 patients underwent conservative treatment and operative treatment was done for 45 patients. We recorded the median APACHE II score, Ranson score, MODS, and SOFA score to assess AP severity and their median scores were 12 (range, 0–32; IQR, 4–15), 4 (range, 0–9; IQR, 3–5), 6 (range, 0–16; IQR, 3–9), and 6 (range, 0–17; IQR, 3–8), respectively. Delirium was found in 49 patients (26.6%).

### 3.2 Serum sFGL2 levels and its association with diseases severity

**Figure 1:**
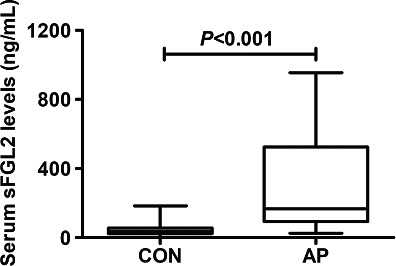
Graph displaying serum soluble fibrinogen‐like protein 2 (sFGL2) levels in healthy controls (CON) and patients with acute pancreatitis (AP)

**Figure 2:**
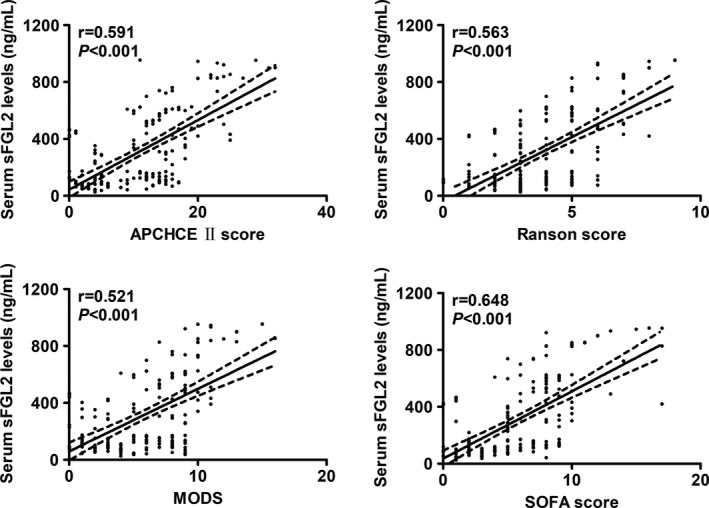
Graph portraying relationship between serum soluble fibrinogen‐like protein 2 (sFGL2) levels and Acute Physiology and Chronic Health Care Evaluation II (APCHCE II) score, multiple organ dysfunction score (MODS), sequential organ failure assessment (SOFA) score in addition to Ranson score in patients with acute pancreatitis

Just as illustrated in Figure 1, serum sFGL2 levels were substantially higher in the patients than in the controls; as regards its correlations with the AP severity, in Figure 2, serum sFGL2 levels were significantly elevated with increasing APACHE II score, Ranson score, MODS, or SOFA score.

### 3.3 Prediction for delirium

**Figure 3:**
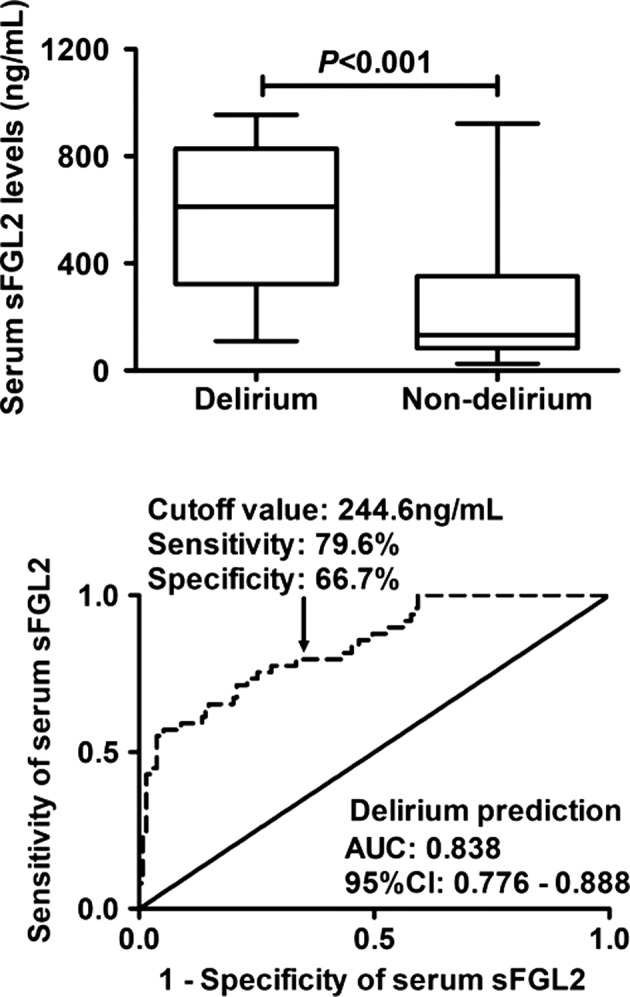
Graph illustrating discriminatory ability of serum soluble fibrinogen‐like protein 2 (sFGL2) levels for patients at risk of delirium based on receiver operating characteristic curve as well as depicting comparison of serum sFGL2 levels between delirium and nondelirium patients. Area under curve (AUC) and the corresponding 95% confidence interval (CI) were estimated

Just as depicted in Figure 3, as opposed to the patients without the development of delirium, the serum sFGL2 levels were substantially increased in those suffering from delirium. In Figure 3, using ROC curve analysis, serum sFGL2 levels markedly distinguished the patients at risk of delirium; meanwhile, we selected 244.6 ng/ml as a cutoff value, and thereby serum sFGL2 levels >244.6 ng/ml predicted the development of delirium with a sensitivity of 79.6% and a specificity of 66.7%. Moreover, its discriminatory ability was equivalent to those of APACHE II score, Ranson score, MODS, and SOFA score (Table 1). In addition, we configured the combined logistic regression model to assess the addictive effect of serum sFGL2 levels to APACHE II score, Ranson score, MODS, and SOFA score. It was demonstrated that the delirium‐differentiating ability of their combination was substantially higher than those of APACHE II score, Ranson score, MODS, or SOFA score alone (Table 1).

**Table 1:** Comparison of the discriminatory ability of serum soluble fibrinogen‐like protein 2 and other variables for delirium after acute pancreatitis

	AUC (95% CI)	*p *Value	*p *Value
sFGL2 levels	0.838 (0.776–0.888)	Ref.	
APACHE II scores	0.878 (0.822–0.921)	0.180	
APACHE II scores^a^	0.933 (0.886–0.964)		0.002^b^
Ranson scores	0.887 (0.832–0.929)	0.127	
Ranson scores^a^	0.937 (0.892–0.967)		0.004^b^
MODS	0.891 (0.837–0.932)	0.082	
MODS^a^	0.942 (0.898–0.971)		0.005^b^
SOFA scores	0.861 (0.802–0.907)	0.476	
SOFA scores^a^	0.930 (0.883–0.962)		<0.001^b^

Note

Area under receiver operating characteristic curve (AUC) was compared using *Z* test. 95% CI indicates 95% confidence interval; sFGL2, soluble fibrinogen‐like protein 2; Ref., reference; APCHCE II, Acute Physiology and Chronic Health Care Evaluation II; MODS, multiple organ dysfunction score; SOFA, sequential organ failure assessment.

a Denotes combination with sFGL2 levels.

b Represents comparison with APCHCE II scores, Ranson scores, MODS, or SOFA scores alone.

Just as listed in Tables 2 and 3, the patients experiencing delirium had higher APACHE II score, Ranson score, MODS, SOFA score, serum C‐reactive protein levels, and white blood cell count, in addition to a higher percentage of surgical treatment and serum sFGL2 levels >244.6 ng/ml. We incorporated those above‐mentioned variables in a multivariate model, and subsequently it was revealed that APACHE II score, Ranson score, MODS, SOFA score, and serum sFGL2 levels >244.6 ng/ml emerged as independent predictors for delirium with OR values of 1.325 (95% CI 1.190–1.475, *p* = 0.007), 2.947 (95% CI 2.020–4.301, *p* = 0.006), 1.956 (95% CI 1.515–2.527, *p* = 0.004), 1.689 (95% CI 1.371–2.081, *p* = 0.003), and 2.607 (95% CI 1.021–6.656, *p* = 0.025) respectively.

**Table 2:** Factors related to delirium after acute pancreatitis using bivariate analysis

	Delirium		*p* value
	Presence	Absence	
Sex (male/female)	33/16	94/41	0.767
Age (years)	54 (39–63)	52 (42–62)	0.869
Body mass index (kg/m^2^)	26.4 (25.1–27.7)	25.8 (23.0–27.8)	0.068
Time between pain and admission (hr)	18.2 (16.0–22.3)	19.1 (12.0–26.2)	0.703
Sample‐collecting time (hr)	20.4 (16.4–23.0)	19.4 (13.5–27.6)	0.261
APACHE II scores	17 (14–23)	9 (4–14)	<0.001
Ranson scores	6 (5–7)	3 (2–4)	<0.001
MODS	9 (8–11)	5 (2–7)	<0.001
SOFA scores	9 (7–10)	5 (2–8)	<0.001
Etiologies (biliary/alcoholic/hypertriglyceremic/others)	17/16/13/3	43/55/24/13	0.465
Treatments (conservative/operational)	14/35	125/10	<0.001
Serum CRP levels (mg/L)	48.9 (34.4–94.9)	23.1 (9.0–41.1)	<0.001
Blood WBC count (×10^9^/L)	16.0 (10.2–17.7)	10.6 (8.3–13.7)	<0.001
sFGL2 levels >244.6 ng/ml	39 (79.6%)	45 (33.3%)	<0.001

Note

APCHCE II indicates Acute Physiology and Chronic Health Care Evaluation II; MODS, multiple organ dysfunction score; SOFA, sequential organ failure assessment; CRP, C‐reactive protein; WBC, White blood cell; sFGL2, soluble fibrinogen‐like protein 2.

**Table 3:** Factors related to delirium after acute pancreatitis using univariate logistic regression analysis

	Odds ratio	95% CI	*p* value
Sex (male/female)	0.900	0.446–1.813	0.767
Age (years)	0.998	0.973–1.024	0.878
Body mass index (kg/m^2^)	1.166	0.998–1.342	0.072
Time between pain and admission (hr)	0.991	0.947–1.038	0.713
Sample‐collecting time (hr)	0.977	0.939–1.017	0.249
APACHE II scores	1.366	1.234–1.513	<0.001
Ranson scores	3.293	2.284–4.747	<0.001
MODS	2.111	1.639–2.719	<0.001
SOFA scores	1.801	1.475–2.198	<0.001
Etiologies (biliary/alcoholic/hypertriglyceremic/others)	0.987	0.698–1.402	0.944
Treatments (conservative/operational)	31.250	12.783–76.394	<0.001
Serum CRP levels (mg/L)	1.022	1.012–1.031	<0.001
Blood WBC count (×10^9^/L)	1.165	1.086–1.249	<0.001
sFGL2 levels > 244.6 ng/ml	7.800	3.570–17.40	<0.001

Note

95% CI indicates 95% confidence interval; APCHCE II, Acute Physiology and Chronic Health Care Evaluation II; MODS, multiple organ dysfunction score; SOFA, sequential organ failure assessment; CRP, C‐reactive protein; WBC, White blood cell; sFGL2, soluble fibrinogen‐like protein 2.

## 4 DISCUSSION

Although it has been reported that pancreatic FGL2 expressions were up‐regulated significantly (Ye et al., 2013, 2014), to the best of my knowledge, this is the first study for determining circulating sFGL2 concentrations and further investigating the relationship between serum sFGL2 levels and the severity in addition to delirium after AP, thereby finding some interesting results as follows: (a) serum sFGL2 concentrations were substantially raised in AP patients, especially in AP patients developing delirium; (b) serum sFGL2 levels were intimately relevant to the traditional parameters for assessing AP severity, namely, the APACHE II score, Ranson score, MODS, and SOFA score; (c) serum sFGL2, which was identified as a categorical variable, emerged as an independent predictor for delirium among AP patients; (d) the predictive ability of serum sFGL2 levels was equivalent to those of the APACHE II score, Ranson score, MODS, and SOFA score; (e) of note, serum sFGL2 levels significantly improved the predictive ability of the preceding severity scale, that is, the APACHE II score, Ranson score, MODS, and SOFA score. Hence, it is deduced that serum sFGL2 levels might rise after AP and its elevation should be closely linked to the increasing severity and the rising risk of delirium after AP, substantializing sFGL2 as a potential biomarker for delirium following AP.

sFGL2 is secreted by T cells and has immunosuppressive activity that prevents maturation of dendritic cells and subsequent T cell activation (Chan et al., 2003; Hancock et al., 2004; Su et al., 2008). Based on the immunosuppressor effect of sFGL2, its overexpression during pathogen invasion can trigger serious complications and immune dysfunction. Expression of FGL2 mRNA was enhanced in the pancreas and peripheral blood mononuclear cells of rats (Ye et al., 2013). In AP patients, FGL2 expression was found in the pancreas and peripheral blood mononuclear cells as well as was demonstrated to be specifically localized to the endothelium of microvessels and inflammatory infiltrative cells in the areas of acute focal, confluent necrosis (Ye et al., 2014). Therefore, such notion could be supported greatly that FGL2 would be released from pancreatic tissues and peripheral blood mononuclear cells after AP. Our data also provided evidence that serum sFGL2 levels should be elevated significantly after AP. Interestingly, adenovirus‐mediated artificial miRNA targeting FGL2 was found to attenuate the severity of AP in mice (Ye, Ding, Chen, & Dong, 2017). Consequently, it was implied that FGL2 might be a promising target for attenuating the AP severity.

Elevated FGL2 expression of pancreatic tissues and peripheral blood mononuclear cells was highly associated with the increasing pathologic severity of pancreatic injury (Ye et al., 2013). Also, in AP patients, there were positive correlations between FGL2 expression in the peripheral blood mononuclear cells and the severity of severe AP, as indicated by scores of Ranson and APACHE II (Ye et al., 2014). The preceding evidence implies that FGL2 might be related to AP progression. The current study found that serum sFGL2 concentrations were substantially and positively correlated with some traditional parameters for AP severity, such as APACHE II score, Ranson score, MODS, and SOFA score. Overall, our data were supportive of the notion that serum sFGL2 levels might have close relation to the AP severity and might be linked to disease progression.

In this study, another intriguing finding was that patients at risk of delirium had higher serum sFGL2 levels. There were two kinds of mechanisms for interpreting origin of sFGL2. The first explanation was that sFGL2 was greatly produced from pancreatic tissues after AP, and through disrupted blood‐brain barrier which clearly exists under organ failure, it damaged brain tissues via inflammatory process, subsequently leading to delirium. The second interpretation was that sFGL2 could be derived from brain tissues injured by other toxic reaction complicated by AP, and via damaged blood‐brain barrier, it could be released into the peripheral blood, thereby leading to high levels of sFGL2. However, those explanations warrant to be validated in future. In order to further analyze the relationship between serum sFGL2 levels and delirium, we screened some variables using univariate analysis and afterwards adjusted for some confounding factors, such as APACHE II score, Ranson score, MODS, and SOFA score. The final data showed that serum sFGL2 levels remained independently related to the risk of delirium. Moreover, we configured the ROC curve and verified the predictive ability of serum sFGL2 levels for AP patients at risk of delirium. Importantly, serum sFGL2 levels showed the similar AUC, compared with those traditional parameters for assessing AP severity, namely, APACHE II score, Ranson score, MODS, and SOFA score. More importantly, serum sFGL2 could substantially improve the predictive ability of APACHE II score, Ranson score, MODS, and SOFA score. Therefore, determination of sFGL2 in the peripheral blood of patients with AP could be beneficial for clinical assessment of delirium.

## 5 CONCLUSIONS

In the current study, we reveal that elevated serum sFGL2 levels are intimately correlated with the AP clinical severity, reflected by the APACHE II score, Ranson score, MODS, and SOFA score, as well as are independently associated with delirium occurring during hospital stay, indicating that serum sFGL2 might serve as a potential biomarker for estimating AP severity and predicting in‐hospital delirium.

## CONFLICT OF INTEREST

The authors have no conflict of interest.
